# An Exoskeleton Design and Numerical Characterization for Children with Duchenne Muscular Dystrophy

**DOI:** 10.3390/bioengineering11111072

**Published:** 2024-10-26

**Authors:** Cristian Copilusi, Sorin Dumitru, Nicolae Dumitru, Ionuț Geonea, Cristian Mic

**Affiliations:** Faculty of Mechanics, University of Craiova, 200478 Craiova, Romania; cristian.copilusi@edu.ucv.ro (C.C.); sorin.dumitru@edu.ucv.ro (S.D.); nicolae.dumitru@edu.ucv.ro (N.D.); ionut.geonea@edu.ucv.ro (I.G.)

**Keywords:** exoskeleton, congenital disorders, walking assistance, numerical processing, kinematics

## Abstract

This research addresses a feasibility study for validating an exoskeleton with kinematic considerations. The designed exoskeleton will be used for children with congenital disorders, especially for a case study characterized by Duchenne muscular dystrophy (DMD). The research core focuses on virtual simulations carried out through the multibody systems theory under an MSC Adams 2012 software environment, with an exoskeleton constructive solution. The designed exoskeleton mechanism is characterized by simplicity, low-cost, and easy-operation features criteria. The results obtained through a numerical processing analysis validate the feasibility study of the proposed prototype.

## 1. Introduction

Children with congenital disorders can face various challenges, including walking difficulties. These challenges can stem from a variety of underlying conditions, affecting different parts of the musculoskeletal or nervous system.

Some common congenital disorders that may lead to walking problems include the following: cerebral palsy, spina bifida, muscular dystrophy, clubfoot (Talpines Equinavarus), developmental dysplasia of the hip, Down syndrome, and congenital limb deficiencies. These are described individually in [1,2,3].

By having insight on muscular dystrophy through common diagnoses from the congenital disorders category, it has been determined that the most common disease is Duchenne muscular dystrophy (DMD). This dystrophy was found to affect 4.78% of a sample of 100,000 newborn children, as reported in [4,5].

DMD is a genetic disorder characterized by progressive muscle degeneration and weakness [6]. This disease is one of the most common forms of muscular dystrophy and primarily affects boys, though in rare cases girls can also be affected. DMD can usually be noticed when a child is between 2 and 6 years old.

Thus, DMD is challenging to diagnose, but with the right care and support, children receiving a diagnosis for DMD can lead fulfilling lives.

DMD management and support can be characterized by a series of procedures and solutions like physical therapy, occupational therapy, surgical interventions, medication and nutritional supplements, early intervention programs, and assistive devices.

Physical therapy is one of the most used procedures in DMD cases and consists of strengthening muscles, improving coordination, enhancing balance (e.g., [7]), and tailored exercises and activities to improve motor skills and walking ability.

Regarding occupational therapy, a child with DMD needs 24-h assistance care from a specialist to assist in developing the skills needed for daily activities and to focus on improving fine motor skills and coordination.

In some DMD cases, there is a need for surgical interventions characterized by procedures to correct anatomical abnormalities, which can improve function and walking ability.

Medication and nutritional supplements are additionally used for managing symptoms like pain or spasticity, or they can be used in conjunction with other therapies.

A child with DMD needs early intervention programs which include special services designed for infants and young children to address developmental delays. Also, these include a team of specialists providing comprehensive care.

Lastly, the use of assistive devices is one of the most common solutions, and it is represented by the use of braces, orthotics, walkers, exoskeletons, wheelchairs, and prosthetics to aid mobility.

Considering the summarized DMD management and support, it is well known that each child’s needs are unique; thus, a multidisciplinary approach involving pediatricians, neurologists, orthopedic surgeons, physical therapists, and other specialists is often necessary to provide care and support.

In recent decades, it was remarked that there has been an evolution of assistive devices, especially those dedicated to children with congenital disorders, which are mentioned here, and the exoskeleton designed for walking assistance and rehabilitation has been described in [8].

Spinal muscular atrophy and cerebral paralysis can be diagnosed through walking solutions for mobility, focusing on the exoskeleton from an engineering perspective [9].

Thus, it can be remarked that an NIH pediatric exoskeleton was designed [10,11]; it is an exoskeleton developed by the US National Institute of Health (NIH, USA) through an intramural research program, which helps to alleviate crouch gait in children with cerebral palsy. It resembles the ReWalk from [12], but the novelty element consists of a knee-powered actuator, which adds additional torque to prevent a crouch gait from forming in the case of children between 5 and 7 years old. It is still in the prototype phase and requires specialists for children’s recovery in a controlled environment.

A similar solution is represented by the Atlas 2030 exoskeleton described in [13,14], which is a robotic system with a complex actuation based on eight actuators designed as a wearable pediatric gait exoskeleton for over-ground walking training. It addresses children with spinal muscular atrophy between 4 and 10 years old. It weighs 14 kg and supports 35 kg. Due to the complexity and large number of actuators, the device is rather expensive and heavy. A main advantage is the additional frame, which supports the body and device weight.

By keeping in mind the terms of command-and-control complexity, there are currently several exoskeletons and rehabilitation systems dedicated to human walking, especially for accomplishing more realistic tasks. In [15], the authors designed an adaptive control system for lower-limb rehabilitation robots that enhances the precision of torque estimation and tracking during rehabilitation exercises. The primary challenge addressed is the accurate and dynamic estimation of interaction torques between the robot and the human user. This is critical in rehabilitation, as it directly influences the effectiveness and safety of the therapy.

Other researchers like [16] designed an adaptive exoskeleton, named AXO-SUIT, in terms of modularity, which can be adapted to various persons. The AXO-SUIT is a modular, full-body exoskeleton designed to assist individuals with varying levels of mobility impairment. This emphasizes the importance of a user-centered design approach, where feedback from potential users and healthcare professionals is incorporated into the development process to ensure that the exoskeleton meets real-world needs. Key features of the AXO-SUIT include its modularity, allowing for customization based on the user’s physical condition, and its adaptability for different rehabilitation goals. The exoskeleton supports both upper- and lower-body movements, making it versatile for different rehabilitation settings. Performance assessments demonstrate that the AXO-SUIT can effectively assist users in tasks ranging from walking to reaching, improving their mobility and quality of life. Also, the researchers propose an advanced algorithm that incorporates adaptive control mechanisms to deal with the variability in user conditions (e.g., strength, injury severity). The system dynamically adjusts to the user’s changing physiological conditions, which enhances the robot’s ability to provide effective support or resistance during rehabilitation. The research shows that this method leads to highly accurate torque estimation and smooth interaction, improving both the user experience and therapeutic outcomes.

Thus, in terms of human-centered design, whether it is the adaptive control in lower-limb robots or the user feedback-driven design of AXO-SUIT could be mentioned, and a key trend is the emphasis on tailoring technology to the specific needs of users. This ensures that the systems are both effective and comfortable, which is critical for user acceptance and successful rehabilitation.

Another rehabilitation system is the Funabot-Suit from [17], which introduces a soft exoskeleton that mimics natural muscle movements through the use of McKibben actuators (artificial muscles). This bio-inspired approach aims to create a more natural interaction between the user and the assistive device by providing kinesthetic feedback, which is often lacking in traditional rigid exoskeletons. The use of McKibben actuators allows the suit to deliver smooth, compliant movements that better reflect human biomechanics, thereby enhancing user comfort and perception of natural movement. The research highlights the potential of this technology to improve motor function in users undergoing rehabilitation by creating a more immersive and naturalistic experience. It also shows promise for reducing muscle fatigue and improving long-term rehabilitation outcomes. Thus, in terms of adaptability and modularity, it can be mentioned that several systems emphasize adaptability, whether through real-time torque adjustment, modular exoskeleton components, or bio-inspired actuators. These features enable more personalized rehabilitation and support a wider range of users and conditions.

Another aspect is given by natural interaction and perception, where the Funabot–Suit and torque estimation approaches highlight the need for more naturalistic interactions between users and assistive devices. This is achieved by mimicking biological movements (as in McKibben actuators) or through accurate, real-time torque adjustments, enhancing the user’s kinesthetic perception and overall experience.

Considering the command-and-control complexity, the research presented in [18] can be mentioned, where a systematic review explores the use of inertial measurement units (IMUs) in motion capture systems designed for rehabilitation purposes. IMUs, which measure acceleration and angular velocity, are becoming increasingly popular in rehabilitation due to their affordability, portability, and ability to capture full-body motion without the need for complex, stationary systems like optical motion capture.

The review from [18] evaluates various IMU-based systems in terms of accuracy, usability, and effectiveness in clinical rehabilitation settings. The findings suggest that while IMUs can provide valuable data for motion analysis, challenges remain in terms of signal processing, noise reduction, and integration with other systems for real-time feedback. However, the technology shows significant promise for home-based rehabilitation and remote monitoring, making it an attractive option for long-term rehabilitation care.

In terms of advancements in sensing technologies, the use of IMUs in motion capture systems highlights the growing role of affordable, portable sensors in rehabilitation. These technologies enable continuous monitoring and feedback, potentially revolutionizing at-home care and long-term rehabilitation strategies.

Focusing specifically on exoskeletons especially designed for children, notable results are reported by the researchers in [19]. Thus, this research accomplishes a feasibility study of a mechatronic gait assistance exoskeleton for DMD children’s therapy. The exoskeleton is designed as an active system to enhance the child’s physical performance.

Considering the muscle weakness of a child diagnosed with DMD, this research concerns an exoskeleton design through virtual simulations and numerical processing carried out for a future prototype used in the case of a child with DMD problems.

Another important aspect of the analyzed devices is the integration of skin-interfacing flexible electronics, which provide a wealth of information about the patient’s health status during experimental tests. Therefore, it is essential to have appropriate sensors for the real-time monitoring of cardiac rhythm, body temperature, and even the angular variations in patients using exoskeletons, as described in [20]. Another research problem that can be found in most of the existent exoskeleton solutions regards the contact force between the foot and the ground. This force plays a crucial role in determining grip and can significantly impact the exoskeleton’s balance and spatial orientation. Researchers from [21] mention the development of force sensors that can be implemented at the level of exoskeleton feet and at the joints level for measuring friction forces, contact forces, and so on. These play an important role in assuring exoskeleton performance, comfort, and safety for patient behavior and can be worn as rehabilitation and assistance systems.

The proposed exoskeleton prototype will be designed based on the previous research reported in [22,23,24], and it will emphasize simplicity, low-cost criteria, and easy-operation features. The simplicity is provided by the mechanism combination, namely Chebysev, pantograph, and Stephenson III’s six-bar mechanisms designed to actuate the hip, knee, and ankle joints of a child with DMD using a single servomotor unit. This mechanism combination enables the movement of these joints with minimal complexity. 

Regarding the low-cost criteria, this will involve the use of additive manufacturing techniques such as rapid prototyping through 3D printing, which allows for the customization of parts for each child with DMD’s specific needs.

The easy-operation features will be achieved through a command-and-control system for a single servomotor unit, which will actuate both legs of the exoskeleton simultaneously in tandem mode.

The research is organized as follows. The first section outlines the challenges and issues represented in the design of exoskeletons and existent prototypes used for walking assistance and rehabilitation in children. The second section presents an experimental analysis of a child’s leg behavior during walking, respectively, a single and complete gait. This was performed on two children, respectively, a healthy one and another diagnosed with DMD. The third section is dedicated to the constructive design of the leg exoskeleton mechanism, starting from a structural scheme and culminating in a virtual model that will be used in the prototyping phase. The exoskeleton virtual model is further used in a kinematic analysis developed with MSC Adams 2012 software, which is presented in the fourth section.

In the fifth section, a comparative study is conducted based on the numerical results obtained in the previous section.

The research concludes with conclusions that validate the feasibility study of the exoskeleton prototype.

## 2. Child Leg Behavior

The child’s leg behavior was experimentally examined during walking activity, covering a complete gait. This experimental analysis was conducted in two key cases using motion analysis equipment, namely CONTEMPLAS [25].

The two cases involve a healthy child and a child with a DMD diagnosis. Both children are four years old and have similar anthropometric data.

The equipment allows for the attachment of reflective markers to the analyzed subjects, which can be tracked by two high-speed cameras during the experimental tests. The equipment is operated using a dedicated software, namely TEMPLO Motion, which can accurately target and track the attached markers.

The experimental analysis setup was established in a specially designed environment of a six-meter-long testing area, where a global coordinate system was defined prior to the analysis to facilitate the calibration of both high-speed motion cameras. 

**Figure 1:**
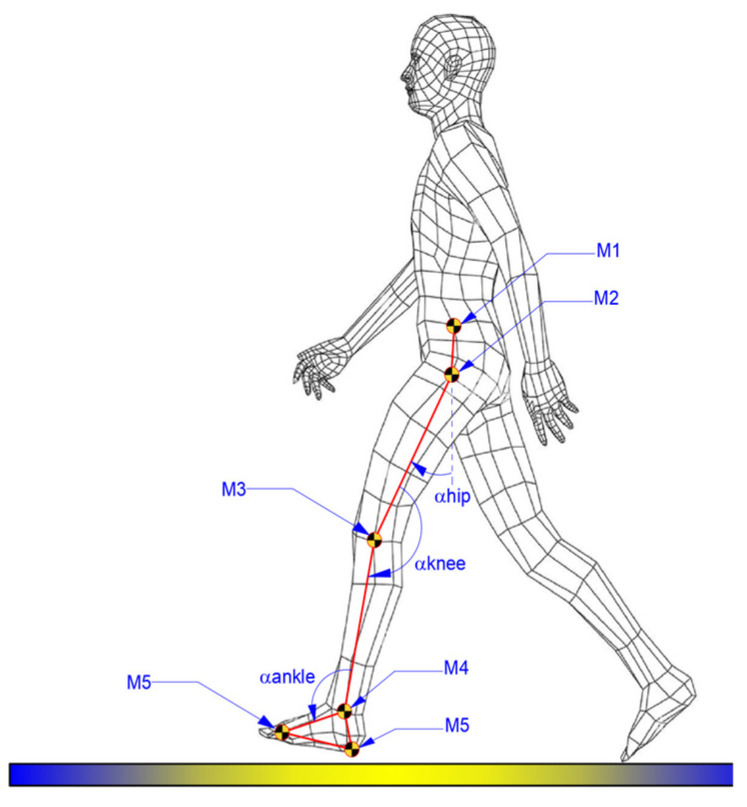
Marker positions and angle identification for the corresponding angular variations.

A physician was present during experimental tests, and the attachment of markers to the children’s locomotion systems was performed based on the physician’s recommendations. For each case, there were attached six markers, as shown in the scheme from Figure 1.

Thus, Figure 1 illustrates the positioning scheme of the markers, and the method for defining the proper angles for each analyzed joint, namely hip, knee, and ankle.

**Figure 2:**
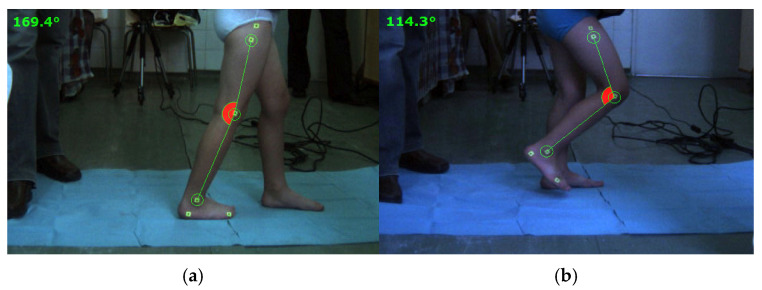
Snapshots during experimental tests of the proposed cases: (**a**)—healthy child; (**b**)—DMD child.

Both experimental analyses were conducted under similar conditions for the right leg of each child, and some snapshots during experimental tests are shown in Figure 2.

**Figure 3:**
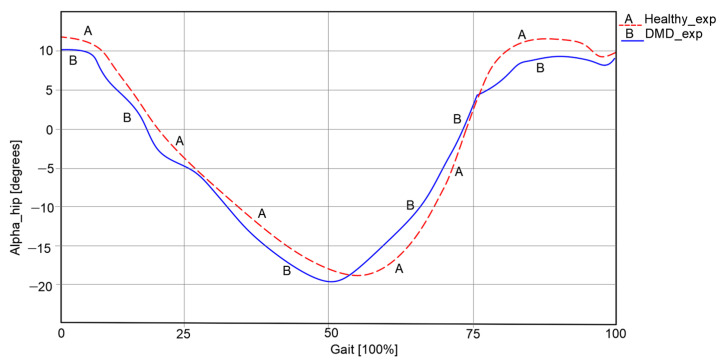
Hip angular variations [°] for a complete gait [100%].

**Figure 4:**
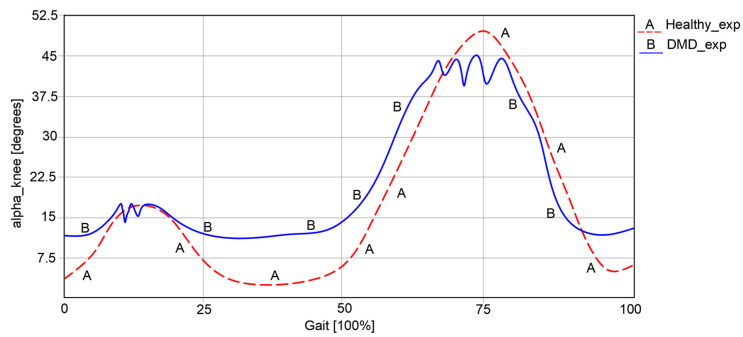
Knee angular variations [°] for a complete gait [100%].

**Figure 5:**
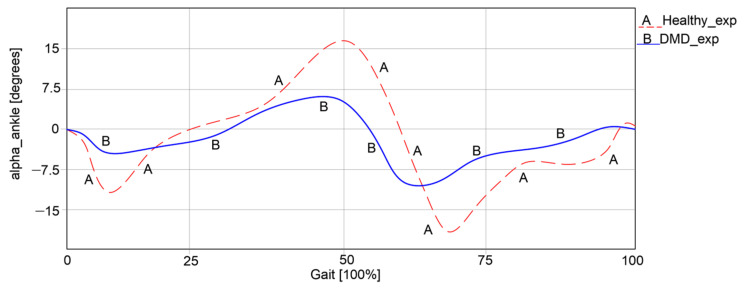
Ankle angular variations [°] for a complete gait [100%].

The results obtained during the experimental test for both cases were characterized by the angular variations in the hip, knee, and ankle joints corresponding to a complete gait cycle of 100%. For the healthy child case, a complete gait cycle was completed in a time interval equal to 1.63 s. These results are presented in comparative analyses, as shown in the graphs reported in Figure 3, Figure 4 and Figure 5. These were also compared with the data from specific literature data according to [26].

For the hip joints, in both cases, as shown in Figure 3, an angular variation between −18.23° and 15.978° can be remarked in the case of the healthy child. For the second case, namely the child with DMD, the path is slightly changed and the limits of the hip joint angle were between −19.92° and 10.39°.

Regarding knee angular variations, as represented in Figure 4, it can be remarked that in the first case of a healthy child, this varies between 5.23° and 49.37°.

In the second case, respectively, an angular variation between 12.32° and 45.33° can be observed for the child with DMD. The obtained curve in the second case has a similar path to the one of a healthy child. However, some spikes were observed, which occurred during the foot’s contact with the ground.

An important observation from the experimental analysis of the case of the child with DMD was the presence of an uncertain instability and a lack of walking coordination.

The ankle joint was also analyzed during walking in both cases, with the results presented in Figure 5. 

Thus, it can be observed that in the first case, an angular variation between −15.73° and 16.28° was obtained. In the second case, the angular variations were significantly lower, ranging between −8.22° and 5.98°. It is obvious that the child with DMD does not use the ankle joint to its full capacity during walking, as the curve differs from the one of the healthy child. Instead, the child often compensates motions by placing more strain on the other joints.

An essential result obtained through this experimental analysis is the acquired anthropometric data in the case of the child with DMD. These data are used in the exoskeleton design as input data for numerical processing. Thus, the femur length L_femur_ = 315 mm; L_tibia_ = 292 mm; L_foot_ = 122 mm. These data have also been used in other research reported in [21].

Additionally, the angular variations obtained from the healthy child case were used in a comparative analysis with the results obtained after the numerical processing of the designed exoskeleton.

## 3. Leg Exoskeleton Mechanism Design

The design of a new leg exoskeleton is based on two key milestones: the exoskeletons developed in previous research and the case study of the child with DMD. Combining these two aspects results in a parametrized exoskeleton specifically tailored to the child with DMD.

Basically, for parametrizing the new leg exoskeleton, it is necessary to first acquire the anthropometric data from the child with DMD, which was achieved in the section on child leg behavior.

On the other hand, insights from previous exoskeleton developments as reported in [20,21] show a significant evolution of the leg exoskeleton designed over time.

**Figure 6:**
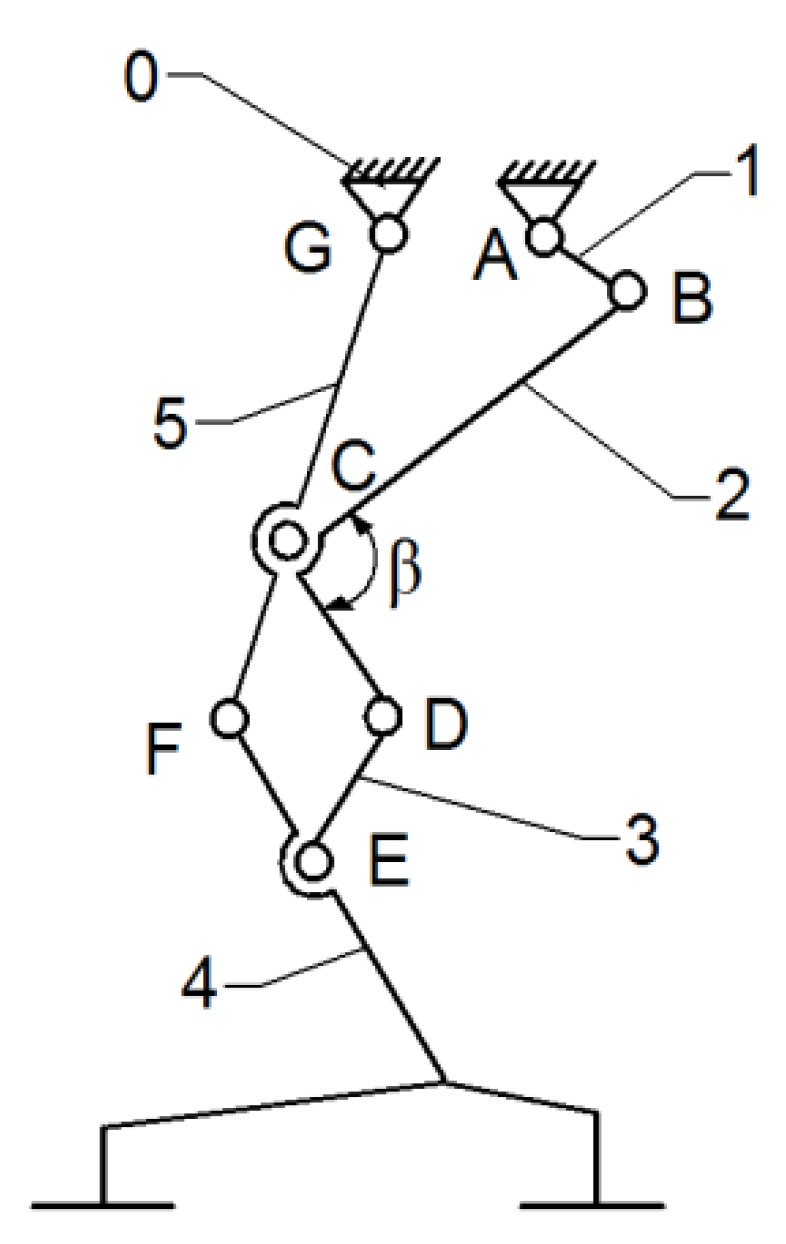
Leg exoskeleton with a Chebysev and pantograph mechanism combination: 0—fixed frame; 1—drive link; 2,3—actuated links; 4—tibia equivalent link; 5—femur equivalent link; A–E—actuation mechanism revolute joints; F—knee equivalent revolute joint; G—hip equivalent revolute link.

Thus, the current exoskeleton platform consists of a combination of two mechanisms, namely the Chebysev and a pantograph one, as shown in Figure 6.

This allows the user to actuate the joints from a single leg, namely the knee and hip joints by the drive link no. 1. The actuated main joints are G, which corresponds to the hip, and F, which properly corresponds to the knee joint.

**Figure 7:**
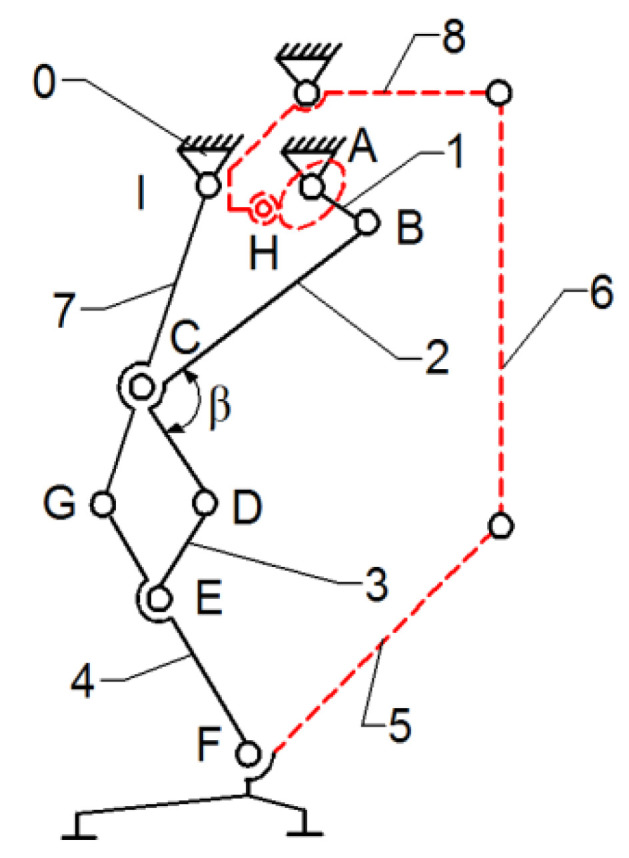
Leg exoskeleton with a Chebysev, pantograph, and a cam mechanism combination: 0—fixed frame; 1—drive link; 2,3—actuated links; 4—tibia equivalent link; 5,6—flexible cables of the cam mechanism; 7—femur equivalent link; 8—cam mechanism actuation link; A–E—actuation mechanism revolute joints; F—ankle equivalent revolute joint; G—knee equivalent revolute joint; H—cam mechanism joint; I—femur equivalent revolute joint.

As a research continuity reported in [22], the insertion of an active cam mechanism inside the presented leg exoskeleton structure can be observed in Figure 7.

In Figure 7, a cam mechanism is schematically shown, actuated in parallel by the drive link no.1. The cam follows a specific motion law, developed to match the ankle joint motion law. It actuates the cam follower H joint, which is connected via flexible links (cable driven) no. 6 and no. 8, and link no. 5, which is equivalent to the foot segment. By incorporating the cam mechanism motion at the ankle joint level, respectively, the F-joint is extended.

The leg exoskeleton working principle, as shown in Figure 7, involves the actuation of all three main joints equivalent to the hip (I joint), knee (C joint), and ankle (F joint) through connection links no. 7 and 4. The Chebysev and pantograph mechanisms actuate the hip and knee equivalent joints, while the cam mechanism actuates the ankle equivalent joint through links no. 5, 6, and 8. Drive link no. 1 will actuate link no. 2 of the Chebysev mechanism and link no. 3 of the pantograph through revolute joints A, B, C, D, and E. Additionally, the cam component of the cam mechanism (H joint) is fixed to drive link no. 1. Due to the particular shape of the cam, which follows the ankle joint motion during a complete gait cycle, the cam follower at the H joint is actuated, transmitting motion to link no. 5, which is connected to the exoskeleton foot segment.

However, this was a temporary solution due to the cam mechanism’s disadvantages, such as large clearances and a lack of control during overloads, which led to an imprecise motion.

**Figure 8:**
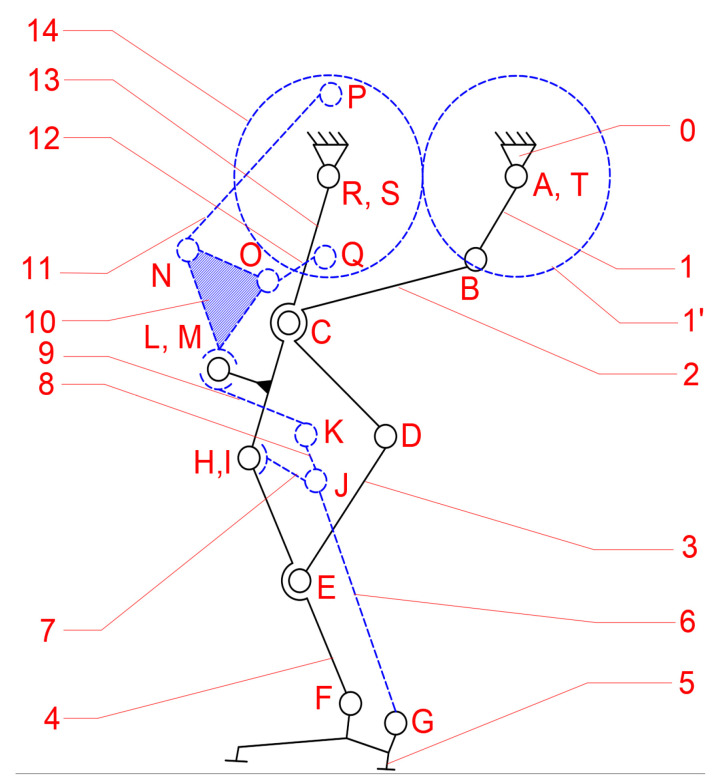
A leg exoskeleton with a Chebysev, pantograph, and a modified Stephenson III six-bar mechanism combination.

Continuing the research outlined in [23], the cam mechanism was replaced with a modified mechanism, namely a Stephenson III six-bar mechanism. A structural scheme of this mechanism is shown in Figure 8.

Although the mechanism presented in Figure 8 appears more complex compared to the previous designs, which may suggest higher manufacturing costs, the mathematical model processed in [23] ensures a precise motion at the ankle joint level and good performance in numerical simulations. Further details on the transition between these mechanisms combination are provided in [23].

According to Figure 8, the proposed leg exoskeleton mechanism combines three mechanism types and consists of the following components: 1—drive link for operating the mechanism of the right hip and knee joints in the exoskeleton structure; 2, 3, 4, 5, 6—kinematic links of the mechanism for operating the hip and knee joints of the right lower limb in the exoskeleton structure; 7, 8, 9, 10, 11, 12, 13—kinematic links of the mechanism for operating the ankle and foot joint of the left lower limb in the exoskeleton structure; 16—rotary electric actuator; 14, 15—gears for operating the ankle joint mechanism of the left lower limb in the exoskeleton structure.

The designed mechanism mimics the structure and movement of a human lower limb to replicate the walking process. It simulates the motion of the knee and hip joints, as well as the foot trajectory, by incorporating segments equivalent to those of the human leg. Link no. 3 plays the structural role of the femur, while link no. 6, through revolute joints H and F, fulfills the functional role of the tibia. The human foot is represented by link no. 5, and its trajectory is an ovoid shape, similar to that produced by human subjects without locomotor deficiencies during walking. Additionally, the variation angles in joints R, H, and F, which correspond to the human hip, knee, and ankle joints, exhibit similar angular variations to those of a person without locomotor deficiencies. It is known that the range of angular variations in these joints can vary from one person to another, or even from one step to another when analyzing the same person. However, within certain limits, an average cycle of these angular variations in the joints in question was considered for the design of the prototype.

The kinematic scheme from Figure 8 represents the combination of the mentioned mechanisms, connected in parallel to operate a single human lower limb. The components shown with solid black lines belong to the mechanism for operating the hip and knee joints, while the components shown with blue dashed lines correspond to the mechanism for operating the ankle and foot joints. Motion transmission from the rotary electric actuator which drives link no. 1 is achieved through a cylindrical gear mechanism with straight teeth, specifically gears 1’ and 14. Gear no. 1 is fixed with drive link no. 1, but gear no. 14 rotates independently from revolute joint R. Gear no. 14 is connected alternatively through revolute joints P and Q to the Stephenson six-bar mechanism, which is identified by links no. 6, 7, 8, 9, 10, 11, and 12. This mechanism actuates the ankle joint, represented by revolute joint F and link no. 5, through revolute joints G, J, H, K, L, M, N, and O, following a specific motion law.

The mechanisms for operating a single human lower limb consist of 15 links connected by 20 revolute joints.

**Figure 9:**
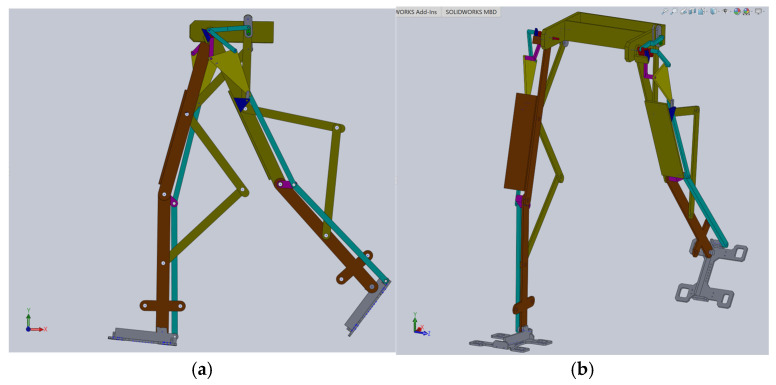
The CAD design of the simplified exoskeleton prepared for kinematic numerical simulations: (**a**)—exoskeleton lateral view; (**b**)—exoskeleton front isometric view.

Based on this structural scheme, a simplified virtual model of the exoskeleton was elaborated in [23], as shown in Figure 9.

The simplified model of the proposed leg exoskeleton was parameterizable, but the examined solution was only suitable for virtual simulations and theoretical studies primarily developed through kinematic and dynamic analyses. It was not suitable for applying to the child with DMD case study.

**Figure 10:**
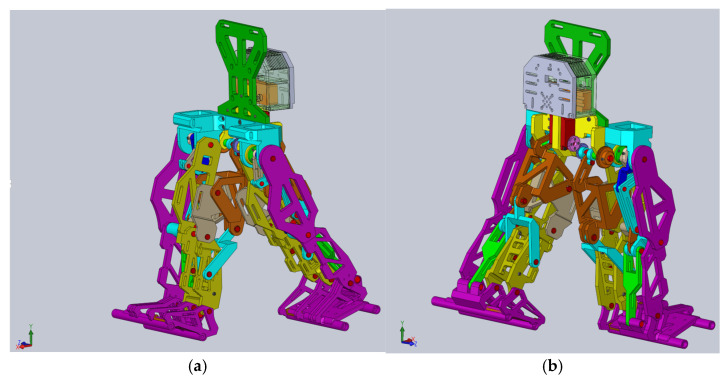
CAD model of the proposed exoskeleton constructive solution: (**a**)—front isometric view; (**b**)—back isometric view.

Using this as a reference point for designing a new leg exoskeleton, a fully parametrized constructive solution was created in SolidWorks 2016, specifically tailored for the DMD child case study. This model, as shown in Figure 10, is ready for virtual testing protocols and numerical analysis.

This constructive solution of the mentioned combined mechanism is fully actuated by a single servomotor.

## 4. Exoskeleton Virtual Simulation and Numerical Processing

This analysis aims to evaluate the leg exoskeleton feasibility design and to characterize its operation performance.

**Figure 11:**
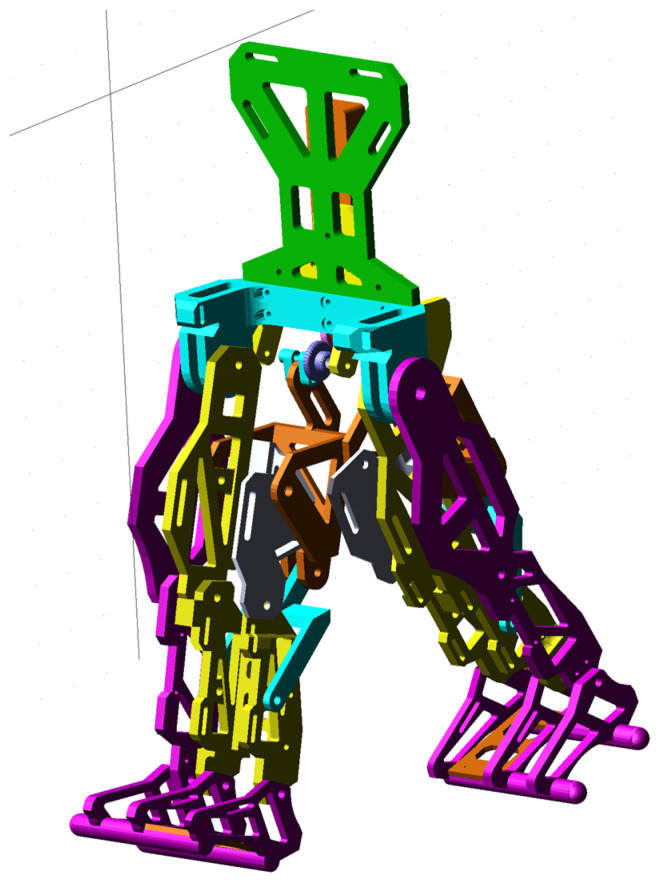
Exoskeleton constructive solution imported in MSC Adams 2012 software for virtual simulations and numerical processing.

The CAD model presented in Figure 10 was fully parametrized for the child with DMD study case. For this, a flexible interface was created for exporting from SolidWorks 2016 software to the MSC Adams 2012 environment. The imported model is shown in Figure 11.

Thus, a dynamic analysis was carried out by following five major steps: defining the proper materials for the links, identifying the center of mass and processing the inertia moments; defining the suitable joints for each leg exoskeleton while considering joint friction; applying loads and establishing the motion law for the appropriate drive link; setting up the proper solver for numerical processing and virtual simulation parameters setup; obtaining the desired results during numerical post-processing. These major steps will be detailed as follows.

At the outset, it can be noted from Figure 11 that the ground was missing, and ground contact would be neglected since the focus was on preparing the final prototype for laboratory experimental tests in future research.

The initial conditions for the proposed constructive exoskeleton model were considered similar to those used in the healthy child experimental analysis. In this case, the exoskeleton was positioned in a biped stance similar to that captured during the complete gait experimental analysis, as seen in Figure 11.

For the first step, in the MSC Adams 2012 environment, the exoskeleton links were defined as made from ABS-plastic with proper mechanical properties similar to those used in 3D-printing processes, namely: tensile strength of 30.2 MPa, tensile modulus of 2.82 MPa, flexural strength equal to 68.7 MPa, flexural modulus of 2.2 MPa, heat deflection temperature equal to 82° Celsius. In the case of pinpoints, these were considered to be made from stainless steel with a Young’s modulus equal to 2.1 × 10^5^ MPa and a Poisson ratio of 0.28.

**Figure 12:**
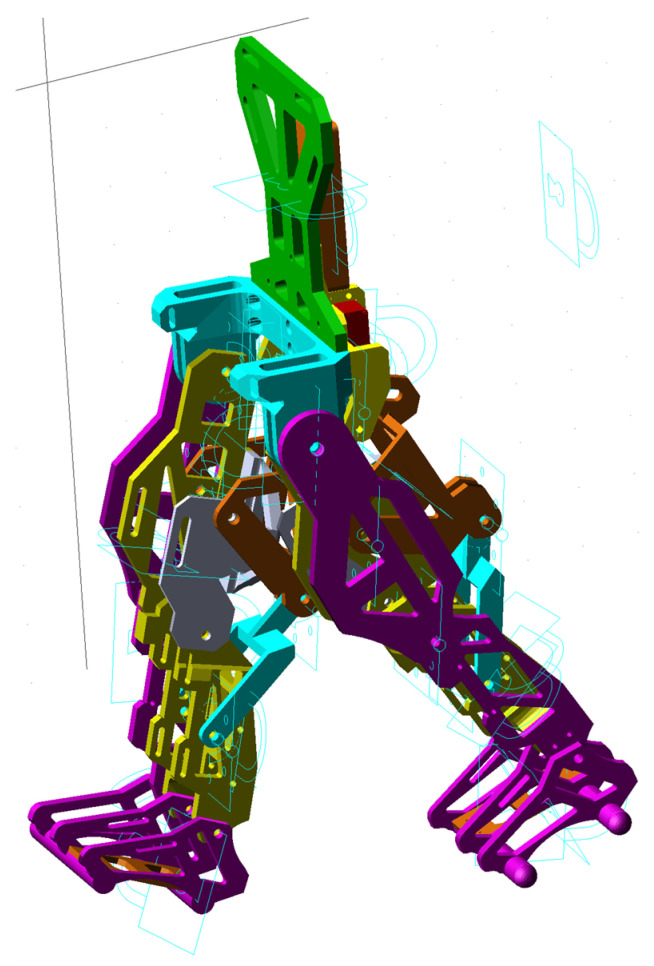
Exoskeleton with the proper joints defined in the MSC Adams 2012 environment.

The next step involved defining appropriate joints to connect the mobile links of the entire exoskeleton linkage. A total number of 13 revolute joints and 21 fixed joints were defined, as shown in Figure 12. The entire structure was considered fixed in space by constraining all DoFs, starting with the equivalent pelvic component.

For these revolute joints, the friction phenomena were defined with the following parameters: static coefficient of 0.012; dynamic coefficient equal to 0.0055; pin radius of the ball joints set at 2.5 mm; stiction transition velocity equal to 0.11; maximum stiction deformation set to 0.01.

The third major step was to define the motion law for the appropriate drive link and load application. Thus, the motion law was set as a function depending on time, based on the following relation:

T_drive-link_: = −60 × time; (1)

**Figure 13:**
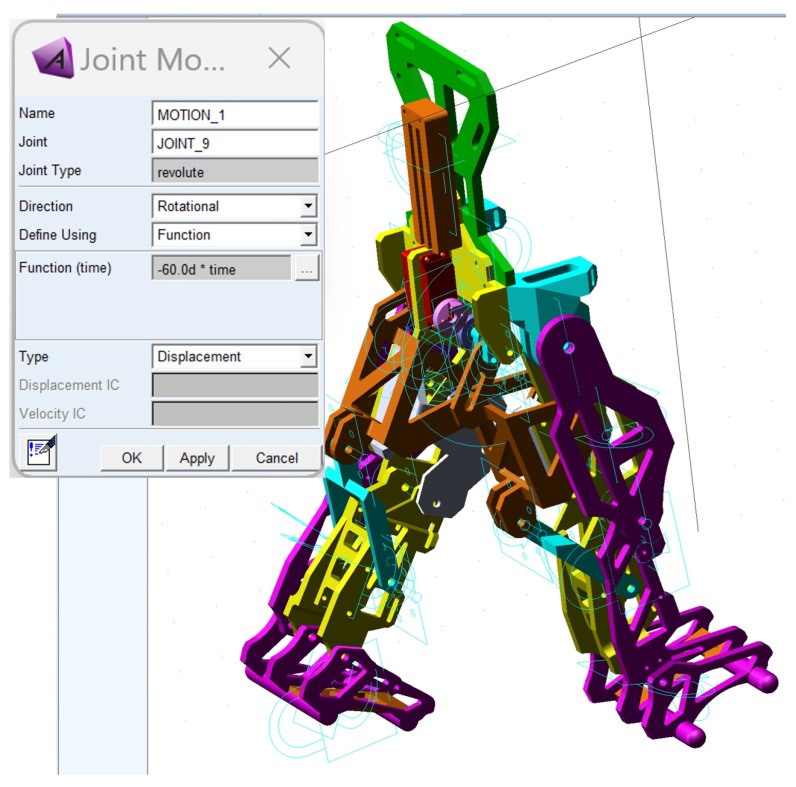
Defining the exoskeleton proper loads under MSC Adams 2012 software.

The load was considered as given by the child with DMD’s weight, which was acquired during the experimental tests, respectively, F_DMD_ = 15.8 kg (154.998 Newton). This was applied in the center of mass of the exoskeleton pelvic component according to Figure 13.

Gravity was also considered as a force acting on the Y-axis, characterized by a negative value.

The dynamic analysis setup was completed in the fourth step of this analysis, using the GSTIFF solver which has an error tolerance of 0.0015.

A walking simulation was computed for a complete gait of the exoskeleton, and this was set to 1.63 s, which corresponds to the time interval recorded during the experimental analysis of the healthy child.

**Figure 14:**
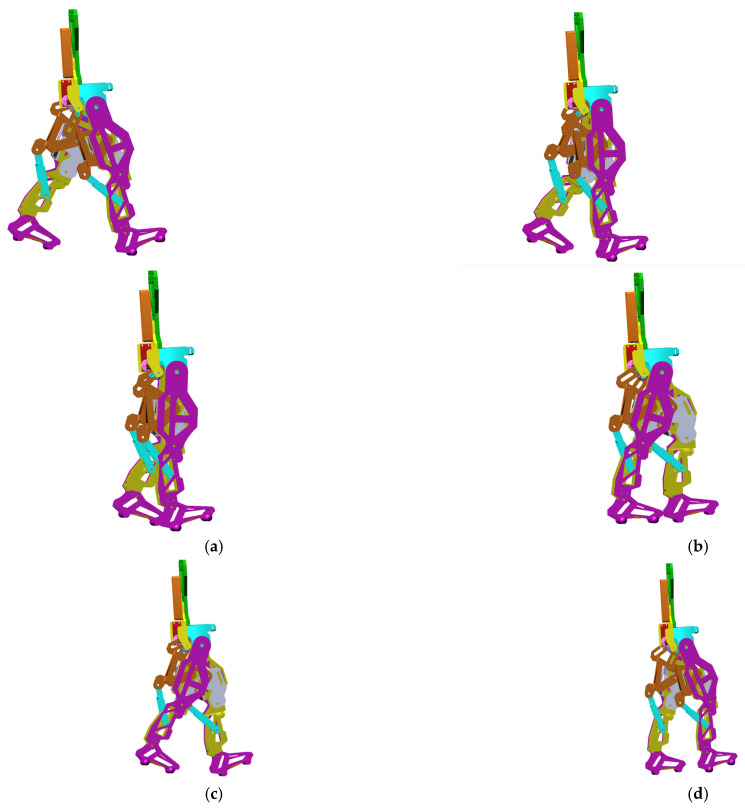
Screenshots during exoskeleton virtual simulations and numerical processing: (**a**)—caption during exoskeleton gait virtual simulations, 25% from a complete gait 100%; (**b**)—caption during exoskeleton gait virtual simulations, 70% from a complete gait 100%; (**c**)—caption during exoskeleton gait virtual simulations, 90% from a complete gait 100%; (**d**)—caption during exoskeleton gait virtual simulations, 100% from a complete gait 100%.

The obtained virtual simulations are presented as a sequence in the screenshots shown in Figure 14, corresponding to a complete gait.

During the virtual simulations and subsequent results’ numerical processing, a series of data and diagrams were generated, illustrating the evolution of various kinematic and dynamic parameters, such as angular displacements, speeds, and accelerations variations in specific links and points, and the reaction forces in the case of the targeted main joints, respectively, the hips, knees and ankles.

To present the results in the final major step, a post-processing interface was created to transfer the data to LS-Dyna R 7.1 software. This transfer aimed to perform a comparative analysis between the experimental results obtained for the healthy child and those derived from the numerical processing of the exoskeleton virtual model. The LS-Dyna R 7.1 software also enables the user to assess the accuracy of the collected data.

**Figure 15:**
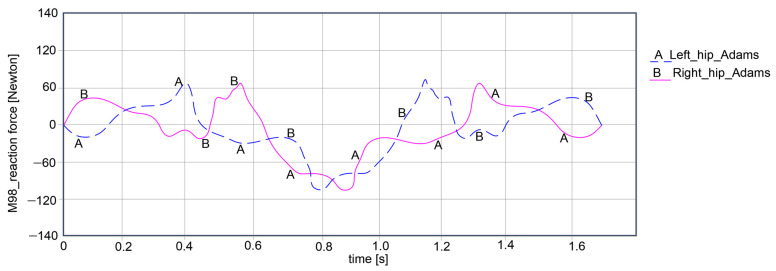
Computed reaction force at the hip joint level of the exoskeleton legs during simulations in Figure 14.

**Figure 16:**
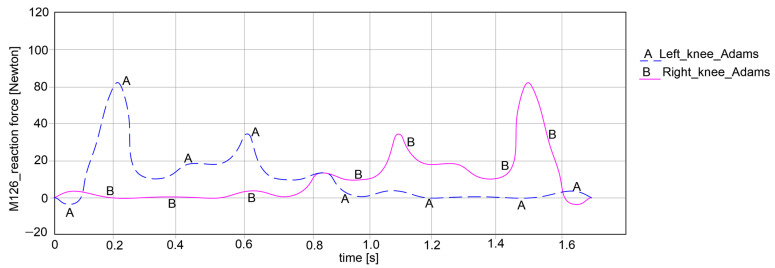
Computed reaction force at the knee joint level of the exoskeleton legs during simulations from Figure 14.

**Figure 17:**
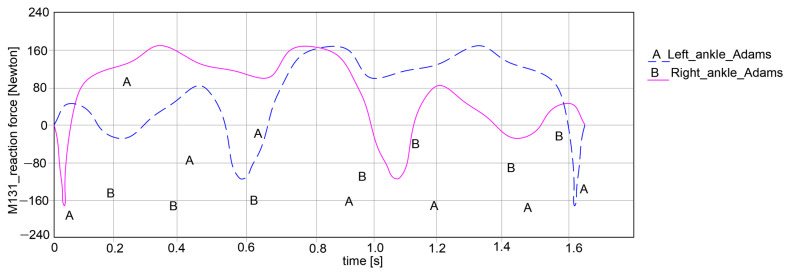
Computed reaction force at the ankle joints level of the exoskeleton legs during simulations of Figure 14.

The key research findings include the reaction forces for the hip, knee, and ankle joints for both exoskeleton legs, as shown in the plots reported in Figure 15, Figure 16 and Figure 17.

Thus, the computed reaction forces for the hip joints show symmetrical curves for both joints, respectively, the left and right, as observed in Figure 15. For the left hip, the reaction force amplitude is equal to 178.939 Newton, varying between 68.385 Newton and −110.554 Newton. For the right hip, the reaction force varies between 68.228 Newton and −110.238 Newton, resulting in an amplitude of 178.466 Newton.

By looking at the knee joints in Figure 16, it can be noted that both paths exhibit symmetrical behavior. For the left knee, the force varies between −5.105 Newton and 86.224 Newton, which means that it can obtain an amplitude of 91.329 Newton. Considering the right knee joint, the values are closer to those of the left knee joint, varying between −4.928 Newton and 85.772 Newton, which yields an amplitude of 90.7 Newton.

In the case of the ankle joint, as in the graph in Figure 17, it can be noted that the left ankle joint shows a variation in reaction force between −164.525 Newton and 162.333 Newton, resulting in an amplitude of 326.858 Newton. Similarly, for the right ankle joint, there is a variation between −163.584 Newton and 162.017 Newton, with a proper reaction force amplitude of 325.601 Newton.

As a general remark regarding the results presented in Figure 15, Figure 16 and Figure 17, it can be summarized that the maximum amplitude of the reaction forces was observed in the analyzed ankle joints. This is given by the weight of the exoskeleton components and the loads applied at the pelvic area, which are influenced by the weight of the child with DMD, specifically 15.8 kg (154.998 Newton).

**Figure 18:**
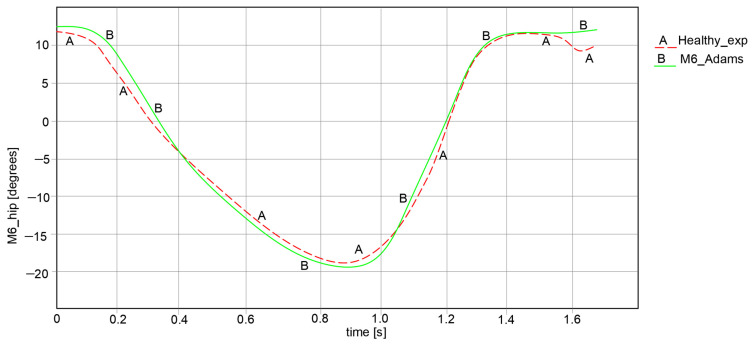
Comparison of analysis results for hip motion law [°] for a complete gait: A—experimental analysis; B—Adams exoskeleton simulation.

**Figure 19:**
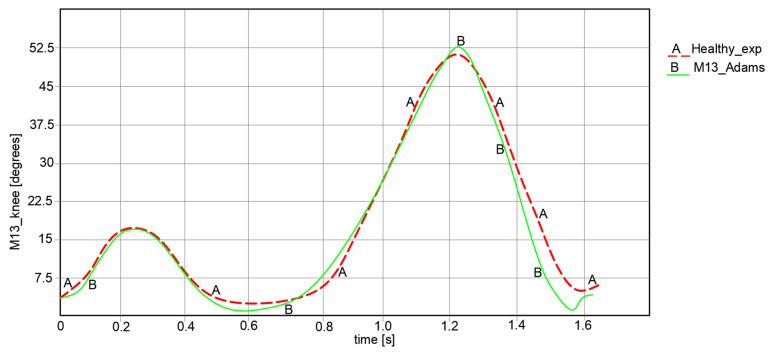
Comparison of analysis results for knee motion law [°] for a complete gait: A—experimental analysis; B—Adams exoskeleton simulation.

**Figure 20:**
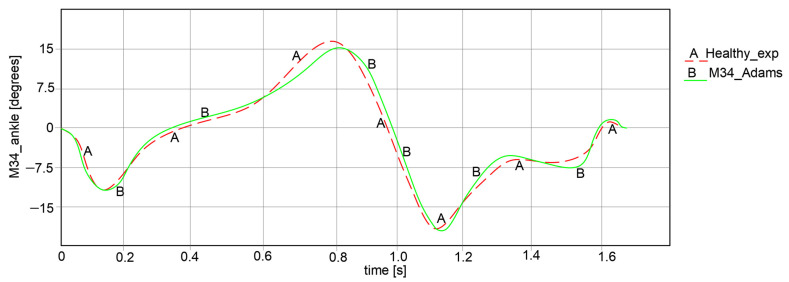
Comparison of analysis results for ankle motion law [°] for a complete gait: A—experimental analysis; B—Adams exoskeleton simulation.

For the comparative analysis, angular variations in the hip, knee, and ankle joints were collected for each leg of the exoskeleton, as can be observed in Figure 18, Figure 19 and Figure 20. The maximum and minimum values are summarized in Table 1, where the calculated accuracy is highlighted. This comparative analysis was performed only for the right limb of the healthy child and the exoskeleton leg. Similar results were obtained for the left limb in both cases, although those values were in the opposite direction.

Considering the comparative analysis of the numerical results presented in the graphs in Figure 18, Figure 19 and Figure 20, it can be remarked that the reported trajectories are very similar between the experimental analysis of the healthy child and the virtual simulations of the elaborated exoskeleton prototype.

Thus, in Figure 18 the hip motion law ranges between −18.334° and 16.078° for the case of the analyzed healthy child, while for the exoskeleton it varies from −20.05° to 14.198°.

For the knee joint comparison, as shown in the graph in Figure 19, the knee motion law varies from 5.325° to 50.12° in the case of the analyzed healthy child, and for the simulated exoskeleton this varies between 4.228° and 52.525°.

A similar comparative analysis was conducted for the ankle joint, as shown in the graph from Figure 20. In this graph, it can be noted that the ankle motion varies from −16.527° to 15.772° for the analyzed healthy child case. For the exoskeleton ankle joint, the motion law is similar to the previous one, with a variation from −16.108° to 15.009°.

By summarizing the obtained results, the accuracy of the considered data was automatically calculated and reported in Table 1. From this table, it can be observed that the highest accuracy was obtained for the knee joint with a value of 1.74%. The lowest accuracy value was obtained for the ankle joint, namely 1.92%.

The average accuracy was 1.81%, which is below 2%, making the results quite acceptable.

These results demonstrate that the future prototype is highly precise in replicating the motions of the DMD child case study, and that it will function as a typical subject, improving the locomotion system performance of the child with DMD.

**Figure 21:**
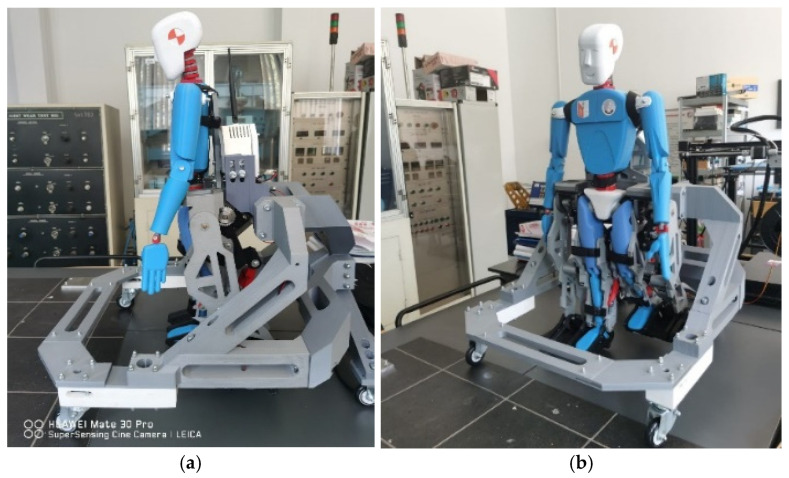
Exoskeleton prototype elaborated for the DMD child case study: (**a**)—exoskeleton lateral view; (**b**)—exoskeleton front isometric view.

A prototype was manufactured using additive engineering techniques, specifically 3D scanning and 3D printing components by using light materials, especially ABS. Figure 21 shows the obtained prototype, which is ready for future laboratory tests.

## 5. Conclusions

This research validates a new design solution for an exoskeleton dedicated to case study of a child with DMD. The exoskeleton incorporates three main mechanisms: a Chebyshev, a pantograph mechanism, and a modified Stephenson II six-bar mechanism. Together, these mechanisms can actuate three main joints of a human lower limb by using a single actuator.

Although the proposed design has a complex structure, it meets the criteria of low cost and easy operation features by using a single drive link as the actuating component. Moreover, this design represents a continuity of previous mechanisms that were designed in earlier research. The peculiarity of the proposed exoskeleton lies in the combination of the mentioned mechanisms, which allows for motion transmission even at the ankle joint level. The proposed constructive exoskeleton design was characterized through virtual simulations and numerical processing analysis using engineering software like MSC Adams 2012 and LS-Dyna R 7.1 software, with models tailored to meet design specific criteria. The obtained results outline the exoskeleton’s functionality, particularly in a case study, respectively, of a child with DMD, and validate the proposed solution for elaborating a prototype using additive engineering techniques and tools. This prototype aims to assist the child during walking. As future directions of the elaborated prototype include expanding the research to a group of several children with DMD problems, improving the contact between the exoskeleton and the child skin and tissue, (addressing the challenge of using the rigid body contact with a deformable body), and at last incorporating force sensors, encoders to monitor the forces at the exoskeleton ground contact, and joints friction forces during angular variations.

## Figures and Tables

**Table 1:** Summary of comparison of results in the graphs from Figure 18, Figure 19 and Figure 20.

Item	Experimental Analysis	Virtual Simulations	Accuracy
Hip joint	Max = 16.078 deg Min = −18.334 deg	Max = 14.198 deg Min = −20.05 deg	1.79%
Knee joint	Max = 50.088 deg Min = 5.325 deg	Max = 52.525 deg Min = 4.228 deg	1.74%
Ankle joint	Max = 15.772 deg Min = −16.527 deg	Max = 15.009 deg Min = −16.108 deg	1.92%

## Data Availability

Data are available on request to the authors.
